# Teachers' Implicit Attitudes Toward Students From Different Social Groups: A Meta-Analysis

**DOI:** 10.3389/fpsyg.2019.02832

**Published:** 2019-12-13

**Authors:** Ineke M. Pit-ten Cate, Sabine Glock

**Affiliations:** ^1^Luxembourg Centre for Educational Testing, University of Luxembourg, Esch-sur-Alzette, Luxembourg; ^2^Institut für Bildungsforschung, Universität Wuppertal, Wuppertal, Germany

**Keywords:** implicit attitudes, teachers, bias, meta-analysis, student group, educational inequality

## Abstract

Teachers' attitudes toward their students have been associated with differential teachers' expectations and, in turn, with students' educational pathways. Theories of social cognition can explain the link between attitudes and behavior. In this regard, the distinction between implicit and explicit attitudes is worth to be considered, whereby implicit attitudes are automatically activated when the attitude object is present and guide automatic behavior. In contrast, explicit attitudes infer deliberation and reflection, hence affecting controlled behavior. As teachers often are required to act immediately in situations that do not allow for thoughtful reflection due to time restraints, teachers' implicit attitudes concerning different student groups with shared characteristics, such as gender or ethnicity, may be especially important when considering teachers' behavior in relation to students' educational pathways. This notion is reflected by an increased interest in adopting implicit methodology in the educational domain. Over the last 10 years, several studies have been conducted in different countries, involving in- and pre-service teachers and investigating their attitudes toward different student groups. Estimates of effects have varied and may be affected by sampling bias. To systematically review and integrate data from different studies, this meta-analysis focuses on teachers' implicit attitudes. Following the systematic search of the database and initial screening, 43 articles were identified from which 22, describing 34 studies, were retained for the meta-analysis after further inspection. First analyses revealed an estimated average effect size of 0.56 for implicit attitudes in favor of non-marginalized groups. As there was a large extent of heterogeneity between studies, several moderator variables were investigated. Results showed that the employed implicit measure and stimulus materials as well as the student target group affected the effect sizes. Low or non-significant relationships were reported between implicit and explicit attitudes. Findings are discussed in terms of theory and future research.

## Introduction

In educational systems around the world, students experience disadvantages in school because of the distinct attributes they share. One such attribute concerns students' ethnicity or immigrant background. A common US definition specifies ethnic minorities as people who are grouped by their race or their cultural origin (Phinney, 1996). Research has shown that ethnic minority students lag behind their ethnic majority peers in academic achievement (Haycock, 2001; Dee, 2005; Marx and Stanat, 2012; Ehmke et al., 2013), tend to drop out of school earlier and often without certification (Rumberger, 1995; Coneus et al., 2009), and receive harsher punishments for misbehavior than their ethnic majority peers (Raffaele Mendez and Knoff, 2003; Peguero and Shekarkhar, 2011; Glock, 2016). Teachers greatly influence their students' academic achievement because they interact with the students in the classroom, assign grades, and refer the students to different school tracks. These interactions and decisions concerning grades and progression through school can partly explain the finding that ethnic minority students are overrepresented in special education programs (Sullivan and Artiles, 2011; Irvine, 2012) and more often classified as having special educational needs (SEN). Students with SEN can have different educational needs arising from medical conditions, learning or behavioral difficulties, or social disadvantages (OECD, 2007). These students also do less well-academically and are more likely to repeat a grade (Landrum et al., 2003). Teachers have lower academic expectations for students with learning or behavioral difficulties (Hornstra et al., 2010; Shifrer, 2013; Hafen et al., 2015), which in turn may influence their decision making and hence contribute to educational inequalities. A third group being disadvantaged in educational attainment concerns students who are overweight or obese. These students do not only suffer from social discrimination (Neumark-Sztainer et al., 2002; Warschburger, 2005), perform less well in school (Latinen et al., 2002; Datar et al., 2004; Shore et al., 2008), miss many days in school (Schwimmer et al., 2003; Geier et al., 2007), are more like to be held back a grade (Falkner et al., 2001), and have low prospects for personal growth (Pingitore et al., 1994; Cawley, 2004; Puhl and Heuer, 2009). Again, teachers contribute to these processes and teachers' expectations in particular are discussed to play a pivotal role when it comes to students with such distinct attributes (Jussim and Harber, 2005). Further vulnerable student groups include students from lower income families (Auwarter and Aruguete, 2008; OECD, 2010) and male students in terms of behavior (e.g., Arbuckle and Little, 2004) and language proficiency (e.g., Hopf and Hatzichristou, 1999; Krkovic et al., 2014) and female students in terms of their mathematical and science abilities (e.g., Keller, 2001; Shapiro and Williams, 2012).

In relation to expectations, attitudes might be vital when teachers are confronted with students representing the above introduced student groups. Attitudes are assumed to influence judgments as well as behavior (Olson and Fazio, 2009). Over the last 30 years, the distinction between implicit and explicit attitudes has been taken into account in social psychological research in many domains. However, in the educational context, the consideration of teachers' implicit attitudes is still in its infancy. In the last 10 years, several studies have been published which have provided mixed results. The aim of this meta-analysis is therefore: (1) to provide an average effect size, (2) to investigate potential moderators of implicit attitudes, and (3) to investigate the relationship between teachers' implicit and explicit attitudes.

## Attitudes as Theoretical Construct

Attitudes are defined as the tendency to evaluate a “particular entity with some degree of favor or disfavor” (Eagly and Chaiken, 1993, p. 1). In their multi-component model, Eagly and Chaiken (1993) differentiate three different components, which add to the overall attitude. They define the cognitive component as knowledge and beliefs about the entity, while the affective component is constituted by the feelings associated with the entity. In case the entity is a social group, the cognitive component is compromised by stereotypes (Eagly and Mladinic, 1989), which are defined as generalized knowledge about the traits, attributes, and behaviors the members of a social group share (Smith, 1998). The last component is the behavioral one and consolidates two different ideas. Firstly, according to the self-perception theory formulated by Bem (1972) people may infer their attitudes from observing their behavior toward objects or persons. However, others argue that people's attitudes guide their behavior and in case of negatively evaluated groups, judgment bias or discrimination is likely to occur (Brewer and Kramer, 1985; Eagly and Chaiken, 1993). Hence the relationship between attitudes and behavior is considered bi-directional, whereby the strength of the attitude determines its impact on behavior and susceptibility to self-perception effects (Holland et al., 2002).

Attitudes are assumed to be the result of life-long experiences with the social group in question (Rudman, 2004). The same assumption underlies stereotypes as the cognitive component of attitudes (Taylor and Crocker, 1981). However, some additional factors come into play. In the early socialization processes, children learn and adopt initial attitudes from their parents (Aboud and Amato, 2001), but in time, attitudes change according to children's own experiences. Mostly, attitudes change in a more positive direction due to intergroup contact (Pettigrew, 1998). In the school context, the contact with students with SEN or students from ethnic minorities creates opportunities that might influence attitudes. Research on intergroup contact theory shows that particularly friendships with members from the negatively evaluated groups can change attitudes (Pettigrew and Tropp, 2008). This may be especially relevant for pre-service teachers that may have had opportunities to interact with students from diverse backgrounds, as in the last 20 years classrooms have become increasingly heterogeneous. Although such contact cannot be easily established if somebody chooses to not have contact with the members of such groups (Pettigrew, 2008), teachers may not have much choice as they usually cannot decide about the composition of their students in class. Other factors, which also fit the school context, are increasing the knowledge about different student groups and reducing the anxiety about interacting with members of these groups (Pettigrew and Tropp, 2008). The two factors are clearly related to teachers' professional experience (Berliner, 2001) and to effective teaching (Bransford et al., 2005; Sharma and Sokal, 2015). Hence, teachers' professional experience can make a difference in attitudes.

Another factor concerns the group membership of teachers themselves (although this may not equally apply to all student categories). Such group membership has a great influence on the socialization processes teachers underwent during their lifetime. For example, research concerning ethnicity has shown that ethnic minority children's attitudes already differ from those of children from ethnic majority groups (see Aboud and Amato, 2001, for an overview). These differences can stem from the dialogue with their parents, who often talk about the ethnic differences and social discrimination with their children (Aboud and Amato, 2001), which in turn makes them more sensitive for such ethnic issues. Additionally, research has shown that people, who belong to an ethnic minority group themselves, show a more differentiated view of their own group (Nosek et al., 2002) rather than the in-group favoritism often reported in ethnic majorities (Dasgupta, 2004). Nonetheless, when people from ethnic minorities perceive their group to be threatened by the majority, in-group favoritism occurs (Crocker et al., 1999). To this extent, teachers from ethnic minority groups might have experienced threat and disadvantages in school as ethnic minority students experience today, and therefore have a better understanding of the concerns ethnic minority students worry about (Gay, 2002; Villegas and Irvine, 2010).

## Dual Modes of Attitudes

When talking about attitudes, the distinction between implicit and explicit attitudes should be taken into consideration. While explicit attitudes are suggested to be conscious evaluations and the result of deliberative processes, implicit attitudes are conceptualized as automatic evaluations (Gawronski and Bodenhausen, 2006) that come immediately into mind when the attitude object is present (Fazio, 2007; Olson and Fazio, 2009). Importantly, the distinction taps into the multicomponent model (Eagly and Chaiken, 1993) outlined above. Hence, implicit attitudes reflect the affective component, as they are suggested to be associations between the object and its evaluations (Fazio, 2007), which is taken into account in all implicit methods. The assessed evaluations mirror the feelings and valences related to the object, which are the result of automatic processes (Gawronski and Bodenhausen, 2007). In contrast, the explicit attitudes reflect the cognitive component because these often rely on beliefs about the attitude object (Gawronski and Bodenhausen, 2007).

The distinction is also taken into account in dual process models, such as the MODE (*M*otivation and *O*pportunity as *De*terminants; Fazio, 1990; Olson and Fazio, 2009). This model builds on the implicit-explicit distinction because it assumes that attitudes guide behavior via two different modes. The automatic path suggests that implicit attitudes mainly guides automatic and spontaneous behavior, while the controlled path assumes that the explicit attitudes guide controlled and conscious behavior. Such thoughtful and effortful processes however, can only occur when the cognitive resources are plentiful and people have much time and motivation to extensively reflect on their behavior and attitudes (Fazio, 1990; Fazio and Towles-Schwen, 1999). Therefore, people's motivation and the possibility to reflect influence which process occurs. Nonetheless, this stringent dichotomy cannot be easily maintained, as most of the processes are mixed with automatic and controlled processes and attitudes contributing to behavior (Olson and Fazio, 2009). Moreover, because of the automatic character of implicit attitudes, they are always activated in presence of the attitude object (Fazio, 2001) even when people do have the motivation and the possibility to reflect. Hence, implicit attitudes might contribute even to controlled behavior.

All these processes are of particular relevance in the school context. Teaching is stressful (van Dick and Wagner, 2001); teachers—and novice teachers in particular—feel overwhelmed by all the tasks they are required to fulfill (Anderson and Olsen, 2006) mostly under time constraints (Santavirta et al., 2007). This makes the influence of implicit attitudes on behavior more likely, particularly in situations in which teachers have to manage many tasks simultaneously. Such situations result in cognitive overload which paves the way for implicit attitudes and often leads to spontaneity and automaticity in behavior (Gawronski and Bodenhausen, 2006). Not only teacher behavior and their teaching practices in the classroom can be influenced by implicit attitudes but also teachers' judgments about students. To this extent, research has shown that attitudes are also related to judgments (Fazio, 1993; Fazio et al., 1995). Teachers are not only required to manage plentiful tasks, they are also confronted with distinct student groups in their class. Hence, the mere presence of students who share distinct attributes, activates implicit attitudes which, in turn, have the potential to impact subsequent teachers' judgment processes or behavioral decisions.

## Implicit and Explicit Methods to Measure Attitudes

The distinction between implicit and explicit attitudes does not only hold for the differential theoretical conceptualization, but is also a measurement issue (Hofmann et al., 2005). Obviously, people often are not aware of their implicit attitudes (Fazio, 2007) and this automaticity requires different measurement methods than the consciousness of explicit attitudes. While explicit attitudes are assessed via self-reports such as questionnaires, Likert-scales, or semantic differentials, implicit methods often rely on reaction times (Wittenbrink and Schwarz, 2007). One of the most prominent methods is the Implicit Association Test (IAT; Greenwald et al., 1998), which is based on the assumption of the associative network theory (Wyer and Carlston, 1994). Within such a network, there are nodes and links. The nodes represent different constructs such as ethnic minority students or positive affect, and the different nodes are connected via links, which vary in strength, depending on how closely the different concepts and nodes are interrelated (Smith, 1998). The IAT utilizes these principals in a categorization task. If for instance, the construct and positive affect are strongly linked and interrelated, people should be able to categorize words or pictures representing the construct and positive affect more easily and faster when they share the same response key as when they share different keys or when the construct is paired with negative affect. The IAT is well-established and its validity (Greenwald et al., 2009) and reliability (Hofmann et al., 2005) is extensively documented. Another method, which is often used in empirical studies (De Houwer et al., 2009), is the Affective Priming Task (APT; Fazio et al., 1995). Like the IAT, this method relies on reaction times (De Houwer et al., 2009) and on associative network models, but does not only utilize the links between concepts, but also the idea of spreading activation (Collins and Loftus, 1975). In the APT, objects or constructs are shown, which should automatically activate the corresponding evaluation or affect. The evaluation is still active when people are asked to categorize words as pleasant or unpleasant directly afterwards. Other measure, such as the Affect Misattribution Procedure (AMP; Payne et al., 2005) or the Sorting Paired Features Task (Bar-Anan et al., 2009) rely on similar theoretical frameworks, but have not been used to the extent of the IAT or the APT.

## Research to Date and Aim of the Meta-Analysis

Although numerous studies have focused on teachers' attitudes toward specific student groups, only a few reviews have been published (Sleeter, 2001; Sze, 2001; McCoach and Siegle, 2007; Glock and Kovacs, 2013). Only one of these focused specifically on studies concerning teachers' attitudes employing implicit measures (Glock and Kovacs, 2013). This review highlighted differences between teachers' implicit and explicit attitudes toward students from ethnic minorities or students with special educational needs and argued research should consider both to better understand their differential effects on teachers' classroom behaviors. As there typically is considerable methodological variation between studies, it remains unclear to what extent teachers hold differential implicit attitudes toward different groups of students. Therefore, the primary objective of the current meta-analysis was to estimate the average effect size of the relationship between student groups and teachers' implicit attitudes. In addition, we aimed to investigate variables that may affect teachers' implicit attitudes (moderator variables). We assumed studies using the IAT would yield stronger effects as the IAT—in contrast to the APT—requires participants to explicitly categorize the target. We also expected an effect for the modality of the prime target, as previous research indicates stronger effects for pictures than words (Spruyt et al., 2002). Furthermore, we expected that teachers' implicit attitudes may vary in accordance with the group of students that were considered. Given the fact that students with ethnic minority background are one of the most marginalized groups in educational systems (OECD, 2010), we expected that studies focusing on ethnicity would report stronger effects than studies focusing on other groups of students. In regards to professional status of teachers (pre- vs. in-service teachers), we expected stronger effects in studies involving pre-service teachers, as more positive attitudes have been reported for teachers with more teaching experience and contact with different groups of students (Pettigrew and Tropp, 2008; Glock et al., 2019). Lastly, we expected differences between European and non-European studies, given historical, political, cultural and educational differences between continents in regards to the perception of students from different target groups. In a final step, we considered to what extent teachers' implicit attitudes are related explicit attitudes.

## Methods

### Search Strategy

In order to identify studies focusing on teachers' implicit attitudes toward different student groups, a search within the electronic databases ERIC, PsychINfo, and Web of Science was conducted using the keywords: implicit attitudes AND teach^*^ OR implicit attitudes AND education.

Only publications in English, as the shared scientific language, were considered, even though studies within this domain may have been published in other languages. In addition, the reference lists of identified papers were searched as well as content lists of journals in which more than two of these articles were published (i.e., *Social Psychology of Education* and *Studies in Educational Evaluation*). Only studies published in scientific journals were considered, excluding doctoral dissertations, book chapters, conference proceedings, and reports, to ensure that the studies had been subjected to a relative standardized procedure of peer-review.

### Criteria for Including and Excluding Studies

To be included in the meta-analysis, studies had to be published in English in the last 20 years (1998–May 2019) and quantitative; implicit attitudes had to be measured as dependent variable and to be related to specific student attributes rather than to abstract constructs (e.g., “inclusion”); the implicit assessment had to reflect affective responses (like/dislike; positive/negative; good/bad) rather than perceived attributes (e.g., stereotype based expectations concerning students' behavior). Moreover, participants had to be pre- or in-service teachers; and studies had to provide effect sizes or provide sufficient data within the manuscript to compute these. We excluded studies focusing solely on explicit attitudes, studies measuring implicit stereotypes, studies involving peers or parents, qualitative studies, and studies published in languages other than English or in (edited) books, dissertations, or conference proceedings.

**Figure 1:**
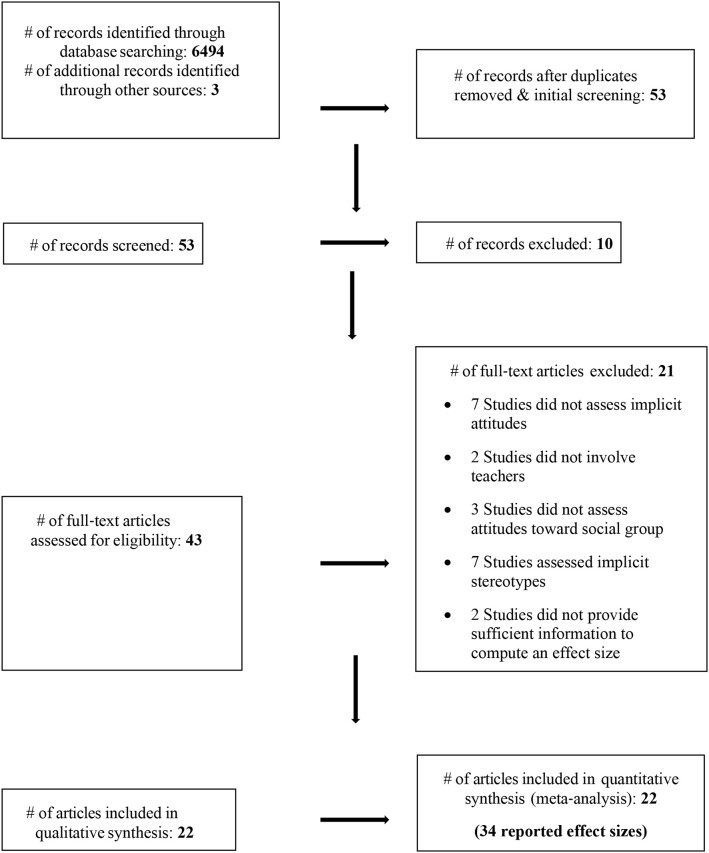
Schematic presentation of selecting studies for meta-analysis (in accordance with PRISMA guidelines; Moher et al., 2009).

Our search identified 6,497 potentially relevant studies. Initial screening and removing duplicates reduced this pool to 53 articles which were subjected to further screening, after which an additional of 10 articles were excluded. After screening the full texts of the 43 articles, an additional of 21 were excluded (please see Figure 1 for specific criteria based on which these articles were excluded). We were able to extract 34 effects from the remaining 22 articles (see Figure 1).

### Data Preparation

For all studies, effect sizes were (re)coded in favor of the non-marginalized groups. More specifically, for all studies, positive *d*-scores reflect negative implicit attitudes toward the marginalized student groups (e.g., students with special needs or from ethnic minorities). Effect sizes were retrieved either as reported in the original manuscripts or computed based on reported descriptive statistics (Means and pooled *SD*), or reported test statistics such as correlations or $\eta_{\text{P}}^{2}$ in accordance with the guidelines of Borenstein et al. (2009) and using an online tool (Lenhard and Lenhard, 2016).

For two studies (Conaway and Bethune, 2015; Krischler and Pit-ten Cate, 2019), multiple dependent effect-sizes related to multiple comparisons were reported. In accordance with the guidelines of Borenstein et al. (2009), these were averaged into a composite score. Some studies reported whole sample or average effect sizes, as well as additional effect sizes for separate social groups (e.g., attitudes toward male and female students from ethnic minorities), in which case only whole sample or average effect sizes were used. For studies that reported different effect sizes for different groups of teachers (e.g., pre- and in-service teachers) these were considered as separate studies (Borenstein et al., 2009; van Rhee et al., 2015) and all effect sizes were considered in the meta-analyses. For one study (Glock and Karbach, 2015), in which three different measures assessing implicit attitudes were compared employing a within-subjects design, we only considered the effect size based on the IAT, as the most commonly used instrument across the studies included in the meta-analysis.

In preparation for the meta-analysis, we noted the student group under investigation, direction of effect, effect size and *SD*_*ES*_, number of participants, and professional status of the teachers, and country of study. If available, we added more detailed information on the participants (e.g., ethnic minority status). If the *SD* of the effect size was not available, we computed the variance based on the reported or estimated correlation, from with the standard error could be derived.

### Statistical Analysis

We conducted the meta-analyses using an MS Excel workbook adapted from Suurmond et al. (2017). In a first step, we conducted a meta-analysis based on the individual effect size, standard error, and sample size. We used a random effects model, which assumes true effects to vary between studies (Borenstein et al., 2009; Field and Gillett, 2010) to estimate a mean effect. In addition, tests of heterogeneity were considered (*Q, p*_q_, *T*^2^, and *I*^2^). In a second step, we conducted moderator analyses to investigate the influence of four variables: country of study (European vs. non-European); assessment method (IAT vs. APT); professional status (pre-service vs. in-service); and student group (ethnic minority vs. other). Although other variables may be important (e.g., characteristics of the participants), such information was not systematically reported resulting in too many missing data points to reliably conduct a moderator analysis. Finally, we summarized results concerning the relationship between implicit and explicit attitudes.

## Results

The data included 22 publications reporting 34 studies. Studies were published between 2010 and 2019. Implicit attitudes concerned students with varying ethnic minority backgrounds (23 studies), students with obesity (five studies), students with special educational needs (three studies), gifted students (two studies), or students from families with low socio-economic status (one study). These studies involved a total of 2,674 in- and pre-service teachers (number of participants per study ranged from 5 to 241). Studies were conducted in Europe (Germany, Italy, Luxembourg, the Netherlands), Asia (Hong Kong) and the United States of America. Most studies assessed implicit attitudes with a variation of the IAT (27 studies), whereas seven used the APT. Materials varied considerably between studies, with 18 studies using student names for the target category or prime and others using student photos (seven studies) or words (e.g., “learning difficulty” for SEN; nine studies). For the attributes, most studies (21 studies) used positive/negative categories, others good/bad (eight studies), or pleasant/unpleasant (five studies). Seventeen studies involved in-service teachers, 16 pre-service teachers, and one study both. Samples included in-service teachers in primary education (five studies), secondary or tertiary education (six studies), or both (five studies). Samples of pre-service teachers were generally mixed (five studies), but some only included pre-service teachers majoring in primary education (one study), secondary education (one study), or special education (one study). For 10 studies (nine involving pre-service teachers), the school type was not specified. Mostly, teachers were considered as a generic group, however, in some studies, they represented specific groups such as Physical Education (PE) teachers (three studies) or teachers with ethnic minority background (three studies). Detailed characteristics of the studies are presented in Table 1.

**Table 1:** Studies included in the meta-analysis.

**No. of** **study**	**References**	**Effect** **size (d)**	**SE**	**No. of participants (N)**	**Student group**	**Scoring direction**	**Professional status participants (school type)**	**Measure**	**Materials**	**Country**
1	van den Bergh et al. (2010)	0.44	0.05	41	Ethnicity: Dutch vs. Turkish/ Moroccan	In favor of students without immigrant background	In-service (primary)	IAT	Target: student name (male) Attribute: words (good/bad)	Netherlands
2	Conaway and Bethune (2015)	0.18^a^	0.04^b^	147	Ethnicity: Caucasian vs. Hispanic or African American	In favor of Caucasian students	In-service (tertiary)	Brief IAT	Target: student name (male and female) Attribute: words (good/bad)	USA
3	Fontana et al. (2013)	2.00	0.03^b^	36	Obesity	In favor of non-obese students	In-service PE^*^ (no data)	IAT (paper pencil)	Target: words (thin/fat) Attribute: words (good/bad)	USA
4	Fontana et al. (2013)	1.44	0.01^b^	140	Obesity	In favor of non-obese students	Pre-service PE^*^ (no data)	IAT (paper pencil)	Target: words (thin/fat) Attribute: words (good/bad)	USA
5	Glock and Karbach (2015)	0.93	0.12	65	Ethnicity: majority vs. minority	In favor of students without immigrant background	Pre-service (different tracks)	IAT	Target: student picture (male) Attribute: words (positive/negative)	Germany
6	Glock and Klapproth (2017)	1.12	0.19	41	Ethnicity: German vs. Turkish	In favor of students without immigrant background	In-service (primary)	IAT	Target: student name (male) Attribute: words (pleasant/unpleasant)	Germany
7	Glock and Klapproth (2017)	0.61	0.12	41	Ethnicity: German vs. Turkish	In favor of students without immigrant background	In-service (primary)	IAT	Target: student name (female) Attribute: words (pleasant/unpleasant)	Germany
8	Glock and Klapproth (2017)	0.31	0.11	41	Ethnicity: German vs. Turkish	In favor of students without immigrant background	In-service (secondary)	IAT	Target: student name (male) Attribute: words (pleasant/unpleasant)	Germany
9	Glock and Klapproth (2017)	0.91	0.12	41	Ethnicity: German vs. Turkish	In favor of students without immigrant background	In-service (secondary)	IAT	Target: student name (female) Attribute: words (pleasant/unpleasant)	Germany
10	Glock and Kleen (2019)	−0.11	0.10	129	Ethnicity: German vs. Turkish	In favor of students without immigrant background	Pre-service with immigrant background (no data)	IAT	Target: student name (male) Attribute: words (positive/negative)	Germany
11	Glock and Kleen (2019)	0.85	0.07	87	Ethnicity: German vs. Turkish	In favor of students without immigrant background	Pre-service without immigrant background (no data)	IAT	Target: student name (male) Attribute: words (positive/negative)	Germany
12	Glock et al. (2013)	0.19	0.03	40	Ethnicity: majority vs. minority	In favor of students without immigrant background	Pre-service (secondary)	Affective priming	Prime: student picture (male) Attributes: words (positive/negative)	Germany
13	Glock et al. (2019)	1.02	0.09	84	Ethnicity: majority vs. minority	In favor of students without immigrant background	Pre-service - high diversity scenario (different tracks)	IAT	Target: student name (male) Attribute: words (positive/negative)	Germany
14	Glock et al. (2019)	0.71	0.10	61	Ethnicity: majority vs. minority	In favor of students without immigrant background	Pre-service -low diversity scenario (different tracks)	IAT	Target: student name (male) Attribute: words (positive/negative)	Germany
15	Glock et al. (2019)	0.81	0.08	104	Ethnicity: majority vs. minority	In favor of students without immigrant background	In-service—high diversity setting (primary and secondary)	IAT	Target: student name (male) Attribute: words (positive/negative)	Germany
16	Glock et al. (2019)	1.04	0.08	127	Ethnicity: majority vs. minority	In favor of students without immigrant background	In-service—low diversity setting (primary and secondary)	IAT	Target: student name (male) Attribute: words (positive/negative)	Germany
17	Glock et al. (2016)	0.59	0.02	51	Obesity	In favor of non-obese students	Pre-service (no data)	Affective priming	Prime: words (thin/fat) Attribute: words (positive/negative)	Netherlands
18	Harrison and Lakin (2018a)	0.19	0.03	197	Ethnicity: mainstream vs. English Learners	In favor of mainstream	In-service (secondary)	IAT	Target: words (English learner/mainstream) Attribute: words (good/bad)	USA
19	Harrison and Lakin (2018b)	−0.12	0.08	71	Ethnicity: mainstream vs. English Learners	In favor of mainstream	Pre-service (different tracks)	IAT	Target: words (English learner/mainstream) Attribute: words (good/bad)	USA
20	Hein et al. (2011)	0.62	0.08	47	Disability	In favor of non-disabled	Pre-service (special education)	IAT	Target: pictures (disabled/non-disabled) Attribute: words (pleasant/unpleasant)	Germany
21	Hornstra et al. (2010)	0.13	0.04^b^	30	Special educational Needs (SEN)	In favor of non-SEN	In-service (primary)	affective priming	Prime: words (dyslexia/neutral) Attribute: words (positive/negative)	Netherlands
22	Kleen et al. (2019)	0.57	0.09	64	Ethnicity: German vs. Turkish	In favor of students without immigrant background	Pre-service—without immigrant background (no data)	IAT	Target: student name (male and female) Attribute: words (positive/negative)	Germany
23	Kleen et al. (2019)	−0.41	0.14	47	Ethnicity: German vs. Turkish	In favor of students without immigrant background	Pre-service—with immigrant (Turkish) background (no data)	IAT	Target: student name (male and female) Attribute: words (positive/negative)	Germany
24	Kleen et al. (2019)	0.26	0.13	38	Ethnicity: German vs. Turkish	In favor of students without immigrant background	Pre-service with immigrant (not Turkish) background (no data)	IAT	Target: student name (male and female) Attribute: words (positive/negative)	Germany
25	Kleen and Glock (2018)	0.94	0.07	160	Ethnicity: German vs. Turkish	In favor of students without immigrant background	In-service (secondary)	IAT	Target: student name (male and female) Attribute: words (positive/negative)	Germany
26	Krischler and Pit-ten Cate (2019)	0.13^a^	0.01^b^	91	SEN	In favor of non-SEN	Pre- and in-service (primary)	Affective priming	Prime: words SEN/Neutral) Attribute: words (positive/negative)	Luxembourg
27	Kumar et al. (2015)	0.25	0.03	241	Ethnicity: Caucasian vs. Arab/Chaldean	In favor of Caucasian	In-service (secondary)	IAT	Target: student pictures (male and female) Attribute (positive/negative)	USA
28	Lau et al. (2018)	0.48	0.01^b^	100	Obesity	In favor of non-obese students	In-service PE^*^ (primary and secondary)	IAT (paper pencil)	Target: words (thin/fat) Attribute: words (good/bad)	Hong Kong
29	Lau et al. (2018)	0.43	0.01^b^	100	Obesity	In favor of non-obese students	In-service non PE^*^ (primary and secondary)	IAT (paper pencil)	Target: words (thin/fat) Attribute: words (good/bad)	Hong Kong
30	Markova et al. (2016)	0.91	0.03^b^	46	Ethnicity: majority vs. minority male	In favor of students without immigrant background	Pre-service (different tracks)	Affective priming	Prime: student pictures (male) Attribute (positive/negative)	Germany
31	Pit-ten Cate and Glock (2018)	0.81	0.07	70	Parental education: high vs. low	In favor of high education level	In-service teachers (different tracks)	IAT	Target: student name (male and female) Attribute: words (positiv /negative)	Netherlands
32	Preckel et al. (2015)	−0.03	0.03^b^	46	Giftedness: gifted vs. average	In favor of average students	Pre-service (no data)	Affective priming	Prime: Student pictures (female) Attribute: words (positiv /negative)	Germany
33	Preckel et al. (2015)	0.04	0.03^b^	45	Giftedness: gifted vs. average	In favor of average students	Pre-service (no data)	Affective priming	Prime: Student pictures (male) Attribute: words (positiv/negative)	Germany
34	Vezzali et al. (2012)	0.69	0.15	5	Ethnicity: Italian vs. immigrant	In favor of non-immigrant students	In-service (primary)	IAT	Target: student name (male) Attribute: words (positive/negative)	Italy

a Composite score.

b Variance computed based on estimated r = 0.25.

* *PE, Physical Education*.

**Figure 2:**
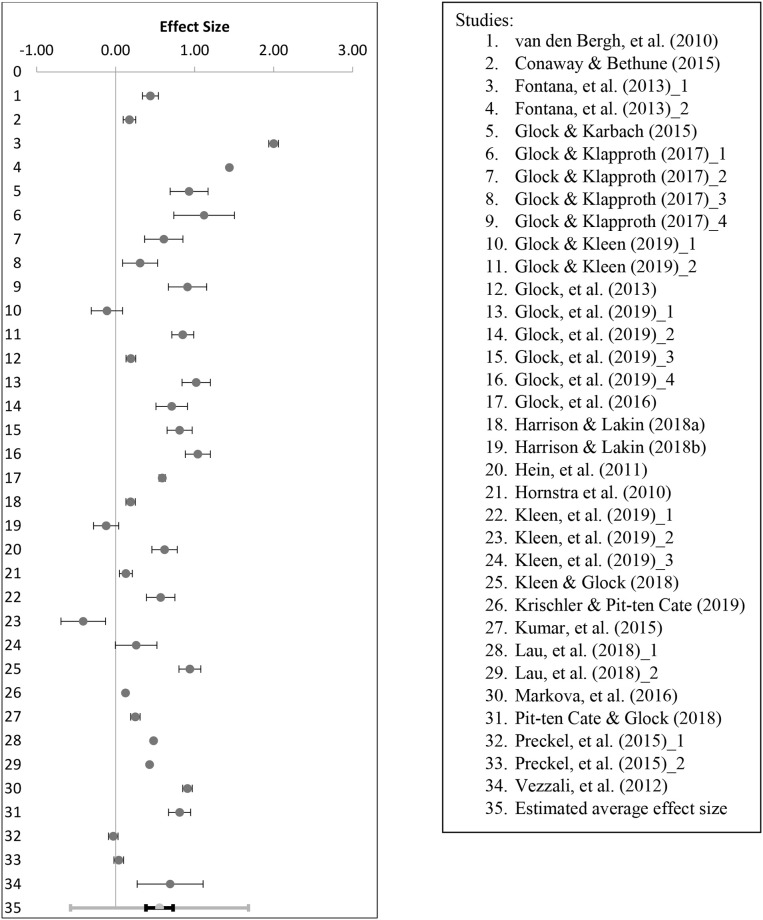
Forest plot for effect size *d* and SE for each study (1–34) in the meta-analysis (Mean *d* = 0.56).

We firstly estimated a standardized mean effect using random effects weights (Borenstein et al., 2009). Assigned weights ranged from 2.68 to 3.01. Figure 2 illustrates the effect sizes for individual studies as well as the estimated average effect size for teachers' implicit attitudes.

The analyses revealed a moderate estimated average effect size (Cohen's *d*) of 0.56, with a 95% confidence interval of 0.38–0.79 (*Z* = 6.61, *p* < 0.001). As the confidence interval does not contain zero, this result indicates that, on average, student characteristics affect teachers' implicit attitudes in favor of the majority group. This result should however be interpreted with caution given the range of observed effect sizes as indicated by the 95% prediction interval.

### Sensitivity

We checked the sensitivity of this estimation by repeating the computations after (a) removing the study reporting the most negative attitudes toward the marginalized group (*d* = 2.00); (b) removing the study reporting the strongest positive attitudes toward the marginalized group (*d* = −0.41); and (c) excluding studies with specific groups of teachers (i.e., PE teachers and teachers with ethnic minority background). Summary statistics are reported in Table 2. Results indicate that the exclusion of certain studies, resulted in only small changes of the estimated average effect size and confidence intervals. However, excluding the studies which included specific groups of teachers (i.e., teachers with ethnic minority background, PE teachers and SEN teachers) reduced the 95% prediction range. Together these findings indicate that the results of the meta-analysis are relatively stable and do not vary substantially as a function of more stringent inclusion criteria.

**Table 2:** Results of the sensitivity analyses.

	**Number of studies *k***	**Estimate of the Mean effect size *d***	***SE* of *d***	**95% confidence interval**		**95% Prediction interval**^**a**^	
				**Lower bound**	**Upper bound**	**Lower limit**	**Upper limit**
Overall effect	34	0.56	0.08	0.38	0.73	−0.57	1.68
Excluded studies							
a) Study 3 (highest effect *d* = 2.0)	33	0.51	0.07	0.36	0.66	−0.53	1.56
b) Study 23 (lowest effect *d* = −0.41)	33	0.58	0.08	0.42	0.75	−0.54	1.71
c) Studies 3 and 23 (highest and lowest effect)	32	0.54	0.07	0.39	0.68	−0.51	1.58
d) Studies 3, 4, 10, 20, 23, 24, and 28 (specific groups of teachers)	27	0.52	0.07	0.38	0.67	−0.04	1.09

a *The prediction interval reflects the range of observed effect sizes (van Rhee et al., 2015)*.

### Heterogeneity

Additional tests were conducted to investigate the degree of heterogeneity. Results showed that there was a significant variation around the estimated mean (*Q* = 13,907.90, *p* < 0.001) and that a very high proportion of the variability in effects was due to differences between the studies (*I*^2^ = 99.76%).

### Moderator Analyses

Results of the sensitivity and heterogeneity tests indicate that there is a need for additional investigation of potential moderators. Therefore, in a second set of analyses, we tested the effect of the measure (IAT vs. APT); materials (student names vs. pictures and vs. words); student group (ethnicity vs. other, ethnicity vs. obesity and ethnicity vs. SEN); country of study (Europe vs. other), and teachers' professional status (pre-service vs. in-service). Results of these analyses, using a random effects model, are presented in Table 3.

**Table 3:** Results of the Moderator analyses.

	**Number of studies *k***	**Estimated average *d***	***SE_***d***_***	**95% CI**	**Predicted range**	**Slope β**	***Z***	***Q***	***df***	***p***	***R*^**2**^**
**Moderator variable**											
Measure											
0) IAT	27	0.63	0.10	0.43–0.83	−0.58–1.83						
1) Affective Priming	7	0.28	0.13	−0.04–0.60	−0.54–1.10	−0.29	−1.59	2.52	1	0.11	8.68%
Materials^*^											
0) Student names	18	0.60	0.10	0.39–0.80	−0.20–1.40						
1) Pictures	7	0.41	0.15	0.04–0.78	−0.58–1.40	−0.23	−2.19	4.81	1	0.03	5.80%
2) Words	8	0.59	0.23	0.05–1.12	−0.85–2.03	0.01	0.05	0.00	1	0.96	0.01%
Student category^*^											
0) Ethnicity	23	0.53	0.09	0.35–0.71	−0.23–1.29						
1) Other	11	0.60	0.19	0.18–1.02	−0.76–1.96	0.24	1.21	1.47	1	0.23	5.62%
2) SEN	3	0.27	0.16	−0.41–0.95	−0.69–1.23	−0.20	−1.79	3.19	1	0.07	4.27%
3) Obesity	5	0.99	0.31	0.12–1.68	0.84–2.81	0.36	1.89	3.57	1	0.06	12.81%
Professional status											
0) In-service	17	0.66	0.11	0.42–0.90	−0.24–1.56						
1) Pre-service	16	0.47	0.13	0.20–0.74	−1.04–1.98	−0.20	−1.13	1.28	1	0.26	3.94%
Country											
0) Europe	26	0.54	0.09	0.37–0.70	−0.19–1.26						
1) Non-Europe	8	0.61	0.26	0.00–1.21	−0.94–2.16	0.06	0.32	0.10	1	0.75	0.37%

* *Moderator analyses were conducted comparing category 0 vs. the other categories (1, 2, 3), respectively*.

The first moderator was the measurement method. We expected differences between studies using the IAT and studies using the APT as previous research has indicated they may measure different constructs (Olson and Fazio, 2003). Even though the impact of the covariate was not significant, observation of the estimated average effect sizes are in line with our assumptions. More specifically, the estimated average effect for studies employing the IAT was moderate and positive, whereas for studies using the APT the estimated average effect is small. In addition to the between measure variation, we also investigated the possible impact of materials used. Results showed a significant effect for the materials used. However, in contrast with our expectations, effect sizes in studies using pictures as target or prime yielded lower effect than studies employing student names or words. The third moderator we investigated was the target group. Results of the analysis did not confirm our assumption as estimated average effects sizes were moderate for both groups and did not differ significantly. However, additional analyses comparing studies focusing on ethnicity with studies focusing on obesity and SEN, respectively, indicated that target groups did influence the effect sizes. Estimated average effect sizes for studies focusing on ethnicity were larger than those in studies focusing on SEN, but smaller than those in studies concerning obesity. Next, we investigated the effect of professional status of the teachers. Results did not confirm our assumption that in-service teachers would have more positive implicit attitudes than pre-service teachers. However, the differences between estimated effects was neither substantial nor significant. Lastly, we investigated the impact of the country in which the studies were conducted. Again, results yielded no substantial or significant differences. Although the impact of the individual covariates was not significant, of the 99.76% (*I*^2^) of between studies variance, 8.68% can be explained by the assessment method (measure), 5.62% by student category (increasing to 12.81% when comparing specific target groups), 3.94% by teachers' professional status, and only 0.37% by country.

### Relations Between Implicit Attitudes and Explicit Attitudes

In a final step we considered relationships between implicit and explicit attitudes (see Table 4).

**Table 4:** Relationship between teachers' implicit and explicit attitudes.

**No. of study**	**References**	**Implicit attitudes**		**Explicit attitudes**
		**Student group**	Effect size (d)^*^	
1	van den Bergh et al. (2010)	Ethnicity	0.44	No significant relationship *r* = −0.06
2	Conaway and Bethune (2015)	Ethnicity	0.18	No significant relationship *r* =^.^16 and *r* =^.^25
3	Fontana et al. (2013)	Obesity	2.00	XX
4	Fontana et al. (2013)	Obesity	1.44	XX
5	Glock and Karbach (2015)	Ethnicity	0.93	XX
6	Glock and Klapproth (2017)	Ethnicity	1.12	XX
7	Glock and Klapproth (2017)	Ethnicity	0.61	XX
8	Glock and Klapproth (2017)	Ethnicity	0.31	XX
9	Glock and Klapproth (2017)	Ethnicity	0.91	XX
10	Glock and Kleen (2019)	Ethnicity	−0.11	Positive relationships
11	Glock and Kleen (2019)	Ethnicity	0.85	Positive relationships
12	Glock et al. (2013)	Ethnicity	0.19	XX
13	Glock et al. (2019)	Ethnicity	1.02	No significant relationship *r* =^.^00 to *r* = −0.01
14	Glock et al. (2019)	Ethnicity	0.71	No significant relationship *r* = −0.09 to *r* = −0.25
15	Glock et al. (2019)	Ethnicity	0.81	No significant relationship *r* = −0.05 to *r* = −0.07
16	Glock et al. (2019)	Ethnicity	1.04	No significant relationship with different dimensions of explicit attitudes *r* = −0.02 to *r* = −0.06
17	Glock et al. (2016)	Obesity	0.59	No significant relationship with the intrinsic or extrinsic motivation to respond without prejudice *r* = 0.28 and *r* = 0.11
18	Harrison and Lakin (2018a)	Ethnicity	0.19	No significant relationship *r* = −0.10
19	Harrison and Lakin (2018b)	Ethnicity	−0.12	Negative correlation *r* = −0.28, *p* < 0.05
20	Hein et al. (2011)	Disability	0.62	No significant relationships with different components of explicit attitudes *r* = −0.21 to *r* =^.^0.07
21	Hornstra et al. (2010)	SEN	0.13	No significant relationship *r* =^.^0.05
22	Kleen et al. (2019)	Ethnicity	0.57	XX
23	Kleen et al. (2019)	Ethnicity	−0.41	XX
24	Kleen et al. (2019)	Ethnicity	0.26	XX
25	Kleen and Glock (2018)	Ethnicity	0.94	XX
26	Krischler and Pit-ten Cate (2019)	SEN	0.13	XX
27	Kumar et al. (2015)	Ethnicity	0.25	XX
28	Lau et al. (2018)	Obesity	0.48	XX
29	Lau et al. (2018)	Obesity	0.43	XX
30	Markova et al. (2016)	Ethnicity	0.91	No significant relationship *r* = −0.09 and *r* = −0.11
31	Pit-ten Cate and Glock (2018)	SES	0.81	No significant relationship *r* = −0.07 to *r* = 0.08
32	Preckel et al. (2015)	Giftedness	−0.03	XX
33	Preckel et al. (2015)	Giftedness	0.04	XX
34	Vezzali et al. (2012)	Ethnicity	0.69	XX

* *Scoring direction in favor of the non-marginalized group*.

Given the debate on the theoretical difference between implicit and explicit attitudes, we checked the studies for the relations between the constructs. Within fifteen studies, correlations between implicit and explicit attitudes were reported. Only three studies reported a significant correlation, whereby in two studies (Glock and Kleen, 2019) more negative implicit attitudes toward ethnic minority students were associated with more prejudiced beliefs, whereas in the other (Harrison and Lakin, 2018b) pre-service teachers that expressed more positive explicit attitudes toward students with ethnic minority background (English learners) had more negative implicit attitudes toward this student group. For the other 12 studies, the association between implicit and explicit attitudes was small and not statistically significant.

## Discussion

The meta-analysis considered studies focusing on teachers' implicit attitudes toward different student groups. Although the IAT was introduced 20 years ago (Greenwald et al., 1998), studies concerning implicit attitudes of teachers only appeared in the last 10 years. From our review of 22 articles comprising 34 studies, we conclude that on average teachers have more negative implicit attitudes toward marginalized groups. The estimated average effect size of 0.56 indicates that this effect is moderate. More specifically, teachers' implicit attitudes differ by half a standard deviation (range 0.38–0.73) in favor of the non-marginalized groups. Several sensitivity checks for the influence of outliers or specific participant groups did not alter the result. Additional results however indicated a large extent of heterogeneity between studies, which implies, the estimated average effect should be interpreted with caution as the effect may differentiate for specific groups of studies. In this regard several moderator analyses were conducted. Results of these analyses indicate that the measure used to assess implicit attitudes may impact the findings. This finding is in line with research showing mixed results concerning the convergent validity of both measures (Olson and Fazio, 2003). The dissociation may stem from differences between the measures. The IAT relies on the associative strength between target categories and attributes. In contrast, the APT is based on the extent to which a prime activates and subsequently facilitates the evaluation of adjectives presented afterwards (evaluative congruent response), whereby responses relate to individual objects rather than to the underlying category. Hence, the IAT and the APT may measure different constructs (Olson and Fazio, 2003) and hence yield different results. In this regard, it is also important to note that the analyzed studies used different stimulus materials within the measures. Where some used student names as a proxy of certain student characteristics, others used pictures or words. Although these variations may not impact the underlying principles of the assessment method, they do produce different effects. More specifically, results of our analyses indicate that using pictures as prime or target may result in smaller effects than when using student names or words. This finding is in contrast with previous findings for the APT, which indicated that using pictures as primes or target produced stronger effects than words (Spruyt et al., 2002). Hence, the influence of the modality of the target or prime may be method specific. Previous research has shown that inconsistent results may stem from the implicit measurement tool used (Glock and Karbach, 2015), especially as different measures apply different categorization tasks (Olson and Fazio, 2003) and hence responses may not only reflect automatic associations but also vary as a function of particular features of the stimuli or categories (De Houwer, 2003). Hence, employing multiple measures of implicit attitudes is recommended in implicit attitudes research.

Variations in findings between studies may also be related to contextual factors of implicit attitudes or its measurement (De Houwer, 2006). Such context sensitivity of implicit attitudes has previously been shown in the context of addictive behaviors (e.g., Glock and Pit-ten Cate, 2015), prejudice (Sherman et al., 2008; Allen et al., 2010), or Antiaging (Gonsalkorale et al., 2014) and should be considered more systematically in future research. Similarly, the context sensitivity of measurements has been demonstrated for both the IAT (e.g., Gonsalkorale et al., 2014) and the APT (e.g., Allen et al., 2010), and hence more advanced measures or models that are able to distinguish between different constructs or components (e.g., Conrey et al., 2005) could further advance this research field.

To this extent, it may also be important to consider the psychometric properties of the implicit measures. Several studies have evaluated the reliability and validity of the IAT (Nosek et al., 2005, 2007; Greenwald et al., 2009). For example, studies have shown that the reliability of the IAT depends on the selection of stimulus materials (Nosek et al., 2007) and may also be related to the scoring algorithm for computing the D-score (Greenwald et al., 2003). In regards to the validity, studies generally support the convergent and predictive validity of the IAT (Nosek et al., 2007; Greenwald et al., 2009), whereas for the construct validity, results are mixed (Nosek et al., 2005; Schimmack, 2019). Therefore, a multimethod, multimodal research design may be preferred to study to what extent people's feelings, thought, or beliefs affect their behaviors, especially when studying sensitive issues (Schimmack, 2019). Given these results, researchers should be aware of differences between and within measures and choose and interpret their method accordingly.

Effect sizes were also affected by the target group under consideration. Estimated average effect sizes varied from small (SEN) to moderate (ethnicity) to large (obesity). It may not be surprising that teachers' implicit attitudes vary based on target group. Teachers' affective responses are based on their experiences and beliefs, which may vary in relation to the group of students under consideration. In this regard, it should be noted that the different samples may have had an additional effect on these findings. For example, in three of the five studies concerning obesity, the sample consisted of PE teachers. These teachers may have different expectations about their students, especially in relation to their ability to do well in sports. In this vein, the weight of a student can more profoundly influence his or her achievement in sport than in the academic subjects. Lau et al. (2018) tested for differences between PE and non-PE teachers but—although implicit stereotypes varied between teacher groups—their implicit attitudes did not. In contrast, for studies focusing on ethnicity, differences between different groups of teachers have been shown, whereby teachers from ethnic minorities (Glock and Kleen, 2019; Kleen et al., 2019) or teachers working in ethnically diverse settings (Glock et al., 2019) show less biased attitudes toward ethnic minority students than teachers belonging to the majority group or working in ethnically homogeneous school settings.

Interestingly, professional status of the teachers (pre- vs. in-service) did not affect the estimated average effect sizes. Previous research indicated that teachers with more teaching experience, especially experience in teaching specific groups of students (de Boer et al., 2011; Glock et al., 2019), show less biased attitudes, which may be explained by increased intergroup contact (Pettigrew, 1998). It may be that the pre-service teachers in the reviewed studies (all published in the last 10 years) already have had opportunities for intergroup contact (Castro and Murray, 2012) or that teacher education programs have specifically prepared them for teaching multicultural (e.g., Sleeter and Owuor, 2011) and diverse (e.g., Sharma and Sokal, 2015) student groups. In addition, intergroup contact may have differential effects on attitudes and beliefs, whereby intergroup contact especially reduces stereotype based prejudice (Weber and Crocker, 1983; Pettigrew and Tropp, 2006). To this extent, some studies in our meta-analysis reported that contact (Hein et al., 2011) or professional status (Krischler and Pit-ten Cate, 2019) resulted in differences in explicit attitudes and beliefs but not implicit attitudes. In contrast, Glock et al. (2019) found that teachers working in heterogeneous schools had less biased implicit attitudes than teachers in homogeneous settings.

The country, in which the studies were conducted, also did not affect the estimated average affect size. This indicates that teachers' implicit attitudes toward certain groups of students are universal (in the western world). This notion is supported by research showing comparable educational inequalities for marginalized groups, especially students from ethnic minorities (Haycock, 2001; Van de Werfhorst and Mijs, 2010; Marx and Stanat, 2012; Peterson et al., 2016).

Findings reported in the studies in this meta-analysis indicate there may be other moderators, such as student gender (Glock and Klapproth, 2017; Kleen and Glock, 2018; Kleen et al., 2019), teacher background or school environment (Kleen and Glock, 2018; Glock et al., 2019; Glock and Kleen, 2019; Kleen et al., 2019) or school track (e.g., Glock and Klapproth, 2017). However, such information was only systematically considered and reported for a very few studies and hence could not be considered in the meta-analysis. Furthermore, it may well be possible that interactions between moderator variables affect effect sizes. However, the program used to conduct the meta-analysis only allows for the assessment of individual moderators (van Rhee et al., 2015) and hence interactions could not be investigated.

Given the theoretical and methodological differences between implicit and explicit attitudes, it is not surprising that all the considered studies reported small or non-significant correlations. These consistent finding validate the distinction between the constructs and indicate both should be considered in attitude research. Only few studies investigate the link between implicit attitudes and teachers' expectations and students' achievement and provide mixed results. More specifically, only in one study (van den Bergh et al., 2010) implicit attitudes were associated with differential expectations, whereas in others this association was not significant (Hornstra et al., 2010; Kumar et al., 2015). Only two studies investigated the association between implicit attitudes and students' actual achievement outcomes (Hornstra et al., 2010; van den Bergh et al., 2010), both supporting the notion that teachers' negative implicit attitudes were related to differences in achievement between student groups. Kumar et al. (2015) were able to show that teachers' with more negative implicit attitudes toward ethnic minority students were less likely to promote respect among the students and resolve interethnic conflicts. Due to the correlational nature of the analysis, cause-effect relationships remain unclear. Other studies have relied on theoretical frameworks to reason about the implications of differential implicit attitudes in favor of non-marginalized groups for students' school trajectories and educational inequalities. These findings indicate that future research should investigate possible links between implicit attitudes and other constructs more systematically to test the suitability of the theoretical models concerning attitudes within the educational domain and to gain better understanding of underlying mechanisms that contribute to the educational inequalities that different groups of students experience.

## Limitations

Some limitations should be noted. First, we only considered English journal articles. The exclusion of other publication formats and publications in other languages may have biased the result and may have resulted in a stronger estimated average effect size. However, due to the observed heterogeneity, the estimated average effect size should already be interpreted with caution. Second, the program used to conduct the meta-analysis does not allow for testing simultaneous or interaction effects of moderators. This may limit the extent to which the complexity of relationships between variables and constructs can be fully understood. As the field develops further and the number of publications increases, future analyses could consider different statistical methods which allow for clustering of effects and enable the investigation of interaction effects (Cheung, 2014; Li et al., 2019). Similarly, the number and details of the included studies only allowed a few moderators to be investigated. As the number of studies increases, additional moderator and subgroup analyses may be conducted. Lastly, not all studies reported sufficient details to draw conclusions on specific characteristics of the sample or to distinguish between subgroups within the sample. Nonetheless, such details may be important to determine variations in implicit attitudes between groups, which in turn, may provide guidance for teacher education programs.

## Conclusion

Although the reviewed research concerning implicit attitudes is characterized by methodological variability, results of the meta-analysis indicate teachers' implicit attitudes are on average moderately biased in favor of non-marginalized groups. The meta-analysis allowed us to investigate different sources of variation and hence provide some directions for future research. Studies in the meta-analysis varied most notably in the use of the implicit attitudes measure, stimulus materials, and target group. Although variation in teachers' professional status was present, this did not seem to affect the results. Instead, it may be specific teacher characteristics (e.g., demographic differences) or differences in school environments that impact their attitudes toward different student groups. Future research should more systematically consider group differences to better understand underlying mechanisms in the formation of implicit attitudes. Of course, methodological differences may also account for differences between studies and future research should consider how differences in design or measurement may affect results. In this context it should be noted that previous research has indicated that implicit attitudes measurement may be affected by both the strength of the association between the object and its evaluations as well as non-associative processes (e.g., self-regulation, context sensitivity). To this extent, more advanced modeling may be used to distinguish between different cognitive processes in the regulation of responses (e.g., Quad model, Conrey et al., 2005; Trip model, Nadarevic and Erdfelder, 2011; ReAL model, Meissner and Rothermund, 2013). Furthermore, one could consider using multimodal measures to further investigate cognitive processes involved in completing implicit measures. For example, Healy et al. (2015) demonstrated that Electroencephalography could be used to examine the extent to responses to implicit measures are resulting from automatic association (activation) or context factors, by relating neural activity to different phases in the IAT. Both the advanced modeling and multimodal approaches could contribute to our understanding of implicit attitudes within the educational area.

The IAT measure may provide the strongest effects, possibly linked to the explicit categorization of groups. For this measure, student names or words may be more suitable for use as targets/primes than pictures. The greatest caveat is, however, formed by the lack of research regarding the association between implicit attitudes, teacher expectations and behavior and, in turn, student outcomes.

## Author Contributions

SG contributed to the conceptualization of the study, the selection of the studies to be included in the meta-analysis, and the theoretical framework of the paper. She edited and approved the final manuscript. IP contributed to the conceptualization of the study, conducted the systematic search, contributed to selection of the studies to be included, and conducted the meta-analysis. She produced the final draft of the manuscript.

### Conflict of Interest

The authors declare that the research was conducted in the absence of any commercial or financial relationships that could be construed as a potential conflict of interest.
